# A Randomized Comparative Study between High-Intensity and Low-Level Laser Therapy in the Treatment of Chronic Nonspecific Low Back Pain

**DOI:** 10.1155/2020/1350281

**Published:** 2020-10-28

**Authors:** Walid Kamal Abdelbasset, Gopal Nambi, Saud F. Alsubaie, Ahmed M. Abodonya, Ayman K. Saleh, Nahla N. Ataalla, Ahmed A. Ibrahim, Sayed A. Tantawy, Dalia M. Kamel, Anju Verma, Samah A. Moawd

**Affiliations:** ^**1**^ Department of Health and Rehabilitation Sciences, College of Applied Medical Sciences, Prince Sattam Bin Abdulaziz University, Alkharj, Saudi Arabia; ^**2**^ Department of Physical Therapy, Kasr Al-Aini Hospital, Cairo University, Giza, Egypt; ^**3**^ Department of Anesthesia and Intensive Care, Faculty of Medicine, Al-Azhar University, Cairo, Egypt; ^**4**^ Department of Surgery, College of Medicine, Prince Sattam Bin Abdulaziz University, Alkharj, Saudi Arabia; ^**5**^ Department of Orthopedic, Faculty of Medicine for Girls, Al-Azhar University, Cairo, Egypt; ^**6**^ Department of Radiological Sciences and Medical Imaging, College of Applied Medical Sciences, Prince Sattam Bin Abdulaziz University, Alkharj, Saudi Arabia; ^**7**^ Department of Physical Therapy, College of Applied Medical Sciences, Hai'l University, Hai'l, Hail, Saudi Arabia; ^**8**^ Department of Physiotherapy, Center of Radiation Oncology& Nuclear Medicine, Cairo University, Giza, Egypt; ^**9**^ Department of Physiotherapy for Integumentary Problems, Faculty of Physical Therapy, Deraya University, Menia, Egypt; ^**10**^ Department of Physical Therapy for Women's Health, Faculty of Physical Therapy, Cairo University, Giza, Egypt; ^**11**^ Department of Physical Therapy for Cardiovascular/Respiratory Disorder and Geriatrics, Faculty of Physical Therapy, Cairo University, Giza, Egypt

## Abstract

**Objectives:**

Chronic nonspecific low back pain (chronic nsLBP) is one of the most common musculoskeletal disorders leading to disabilities and physical inactivity. Laser therapy was used in chronic nsLBP treatment; however, no previous studies have assessed the impacts of high-intensity laser therapy (HILT) versus low-level laser therapy (LLLT) on chronic nsLBP. This study compared the effects of HILT versus LLLT on individuals suffering from chronic nsLBP.

**Methods:**

The study was a randomized control trial. Sixty individuals with chronic nsLBP were enrolled in this study between May and November 2019. All participants were clinically diagnosed with chronic nsLBP. They were assigned randomly into three groups, 20 in each group. The first group received a program of LLLT, the second group received a program of HILT, and the third did not receive laser therapy (control group). Pain severity, disability, lumbar mobility, and quality of life were assessed before and after 12-week intervention.

**Results:**

Both LLLT and HILT groups showed a significant improvement of the Oswestry Disability Index (ODI), visual analogue scale (VAS), lumbar range of motion (ROM), and European Quality of Life (EuroQol) scores (*p* > 0.05), while the control group did not show significant changes (*p* > 0.05). Comparison among the three study groups postintervention showed significant differences in the outcome measures (*p* > 0.05), while comparison between the LLLT and HILT groups showed nonsignificant differences (*p* > 0.05).

**Conclusion:**

There are no different influences of LLLT versus HILT on chronic nsLBP patients. Both LLLT and HILT reduce pain and disability and improve lumbar mobility and quality of life in chronic nsLBP patients.

## 1. Introduction

Chronic nonspecific low back pain (chronic nsLBP) is very common and prevalent among the population worldwide. It is identified as pain remaining more than three months without unknown specific pathophysiology [1]. The incidence of a lifetime of chronic nsLBP is approximated to 84.8% [2]. Chronic nsLBP results in psychological and functional complications and disturbs the quality of life (QoL) [3, 4]. The conventional intervention of chronic LBP includes medical and nonmedical treatment [1, 5].

The underlying causes of the common LBP are exactly undefined [6]. Yearly, ninety-one billion in medical expenditures are spent on back pain with an extra fifty billion indicating indirect costs owing to loss of productivity and payments for disability benefit [7]. One of the significant etiologies of morbidity in adults is LBP [8]. Two-thirds of the individuals are approximately affected several times throughout their lives [9]. It usually causes impairment in the QoL in patients with musculoskeletal system issues [10]. Initial aims of the LBP treatment are to decrease pain, allow patients to continue their activity daily live (ADL), and to improve QoL [5].

Low-level laser therapy (LLLT) is frequently utilized by multiple medicinal sections worldwide; however, the Food and Drug Administration (FDA) has not approved of its indication [11]. The laser emits a monochromatic, nonionizing, noninvasive, polarized electromagnetic, and extremely focused light radiation. LLLT is an effective physiotherapy modality in the management of various musculoskeletal dysfunctions due to its anti-inflammatory, muscle relaxant, analgesic, ligament repairing, tissue revolution, fibroblast exploding, and biostimulant effects [12, 13]. LLLT is usually used to control chronic and acute pain [14]. Another form of laser therapy for controlling musculoskeletal pain is high-intensity laser therapy (HILT). This modality is convenient, noninvasive, and painless, improves joint mobility, stimulates efficiently deepen tissue, and provides anti-inflammation, analgesics, and other useful influences [15, 16]. A previous study found that intense pain can be reduced using pulsed HILT [17]. In addition, it exhibits anti-inflammatory, antiedematous, and analgesic effects for a patient with a pain problem [18]. Moreover, it is able to stimulate areas that are difficult to reach with the LLLT, such as the large and/or deep joints [19].

Several studies found that LLLT is an effective modality for controlling chronic nsLBP [20, 21], whereas others found that HILT is a safe and useful modality in reducing pain in patients with chronic nsLBP [22, 23]. As well, more energy may be transmitted using HILT into the exposed tissues when compared with LLLT [19]. To the best of knowledge, no previous studies have assessed the impacts of HILT versus LLLT on chronic nsLBP. Consequently, the current study aimed to evaluate the different effects of HILT and LLLT on chronic nsLBP, hypothesizing no differences between both forms of laser therapy.

## 2. Materials and Methods

### 2.1. Subjects

This randomized comparative study was conducted in the outpatient physical therapy clinic between May and November 2019. Sixty chronic nsLBP patients were recruited from the orthopedic outpatient clinic at Prince Sattam Bin Abdulaziz University Hospital. All participants were clinically diagnosed with chronic nsLBP using clinical, laboratory, and radiological examinations. They were assigned randomly into three groups using Dacima's Randomization Software, 20 in each group. The first group received a program of low-level laser therapy (LLLT group) plus home exercise training, the second group received a program of high-intensity laser therapy (HILT group) plus home exercise training, and the third group conducted only home exercise training without laser therapy (control group). Randomization was performed by a blinded physiotherapist who was unaware of baseline evaluations. The inclusion criteria were as follows: (a) history of low back pain lasting more than 3 months; (b) age of 25–40 years; and (c) ability to comply with any of the randomly selected treatment programs. The exclusion criteria were neurological defects, abnormal laboratory findings, fracture, spondylosis, spinal stenosis, inflammatory disease, infectious diseases, mental conditions, prior spinal surgery, and pregnancy. Subjects who have received any type of treatment for back pain in the last three months were also removed. Consent forms were obtained from each participant before initiating the study program. This randomized trial has been ethically approved by the Local Ethics Committee of the Physical Therapy Department, Prince Sattam Bin Abdulaziz University (no. RHPT/019/035) according to the guidelines of the 1964 Helsinki Declaration.

### 2.2. Sample Size Estimation

The study power was determined by G ∗ Power 3.1 software with *α* = 0.05, *Z* = 1.95, and power = 0.80. The sample size of 17 participants was required for each group. Hence, this randomized trial has included 20 subjects to account for the dropout rate.

### 2.3. Initial Assessment

Disability, pain severity, range of motion (ROM) of the lumbar flexion, and QoL were examined before and after the treatment program in the three groups by blinded orthopedists that were unaware of the group assignments.

### 2.4. Oswestry Disability Index (ODI)

The ODI is a valid and reliable instrument used to assess the disability level in LBP patients. It comprises 10 items including pain, sleep, walking, carrying, self-help and private life ability, standing, sitting, social and sexual life, and traveling. Every item is scored 0–5 points depending on the real position, with a total score from 0 to 50, and earnest dysfunction means a high score [24].

### 2.5. Visual Analogue Scale (VAS)

VAS is a valid pain intensity measure. Each patient was instructed to place a sign in a 10 cm long part based on the pain severity. It scores 0–10, 0 points to no pain, and 10 points to severe pain. It is commonly utilized to assess lumbosacral pain and it is radiating in the lower limb [25].

### 2.6. Lumbar Flexion Range of Motion (ROM)

The range of motion of the lumbar flexion was assessed using a valid and reliable modified Schober test. This measure is one of the most common measures used to assess the lumbar ROM [26].

### 2.7. Quality of Life (QoL) Questionnaire

The QoL has been measured using EuroQol-5 Dimensions-3 Levels (EQ-5D-3L) [27]. Dimensions include self-care, mobility, pain/discomfort, usual activities, and anxiety/depression with a 0–100 rating scale, where 0 reveals the worst possible health state and 100 is the best possible health state. It is a homogenized tool for determining health outcomes. EQ-5D-3L is a valid and reliable tool, does not necessitate an effort or prolonged timing for filling it, and was recognized in several prior studies to assess QoL in extensive ranges of health conditions. The EuroQol group commended a valid Arabic adaptation of the EQ-5D scale to examine the quality of life. Each dimension involves 3 levels (nothing, slight/moderate problems, and severe/extreme problems) [28].

### 2.8. Intervention

All 60 patients have received home exercise training including strengthening exercise for back and abdominal muscles and stretching exercise for back muscles, at least 2 times per week for 12 weeks. Patients have been instructed to not receive painkillers or nonsteroidal antiseditious medications during the study period [29].

### 2.9. LLLT Protocol

The diode laser device (Chattanooga Group, USA) of gallium-aluminum-arsenide (GaAlAs, infrared laser) with an 850 nm wavelength, 800 mW power, and constant wave with 1 cm spot size was utilized for the treatment procedures. The device of laser was used with a 1 KHz pulsed frequency and duty cycle of 80% with an average energy density of 50 J/cm^2^ for chronic nsLBP for 30 min/session with a total energy of approximately 1200 J, 2 sessions per week for 12 weeks (24 sessions). Each patient has been instructed to lie prone or modified side-lying position during the session. Both the therapist and the participant wore protective goggles for safety during the treatment time.

### 2.10. HILT Protocol

The device of BTL-6000 HILT utilizes a gallium-arsenide diode laser (BTL-6000 laser) and was set manually to biostimulating mode with 12 W power, 150 J/cm^2^ energy, 1064 nm wavelength, and 1 cm^2^ patch radiation diameter zone. Under these cases, the laser was performed with a constant movement. During a session, total energy applied to subjects measured 1200 J, 2 sessions per week for 12 weeks (24 sessions). Application time lasted for 15 min during each session. Each patient has been instructed to lie prone or modified side-lying position during the session. The device probe was vertically situated and moved horizontally on the affected area.

### 2.11. Statistical Analysis

All outcome measures were presented in the form of means ± standard deviations. Changes of the variables among the three groups were assessed using one-way ANOVA, while pre-post changes within each group were assessed using a paired *t*-test. Statistically, all variables were analyzed using SPSS (v.25, IBM Corp., Armonk, NY, USA). The significant level was considered at *p* value ˂0.05.

## 3. Results

**Figure 1:**
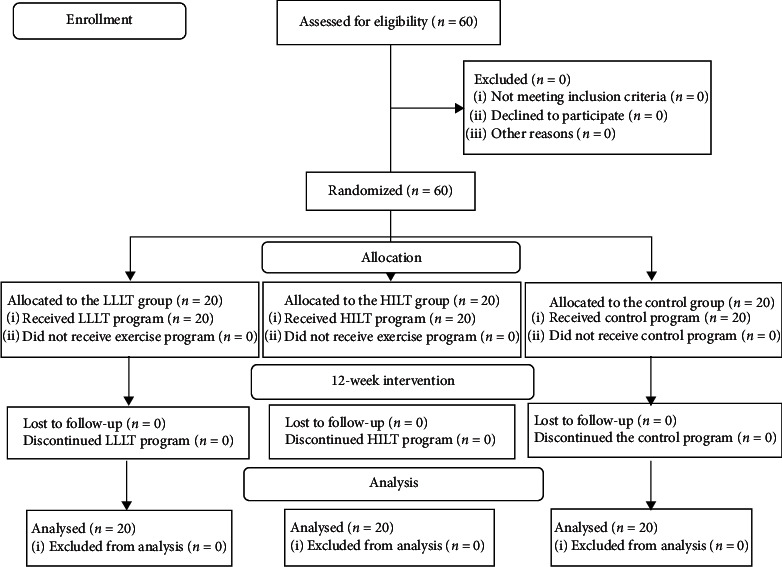
The flowchart of the study.

All the sixty patients have completed the twelve weeks of the study program as described in the flowchart of the study (Figure 1). Baseline characteristics showed no significant differences among the three study groups (age, *p*=0.651; gender, *p*=0.836; BMI, *p*=0.642; LBP duration*p*=0.961; education level, *p*=0.913; occupation, *p*=0.981; and marital status, *p*=0.506) as demonstrated in Table 1. Outcome measures showed no significant differences between groups preintervention (ODI,*p*=0.726; VAS, *p*=0.534; lumbar ROM, *p* = 0.623; and EuroQol, *p* = 0.463). LLLT showed significant improvements of ODI, VAS, lumbar ROM, and EuroQol scores postintervention (*p* < 0.001) and also HILT showed significant improvements of ODI, VAS, lumbar ROM, and EuroQol scores postintervention (*p* < 0.001), while the control group showed nonsignificant changes (*p* > 0.05) as shown in Table 2. Comparison between the three study groups postintervention showed a significant difference in all outcome measures (*p* < 0.001), while comparison between the LLLT and HILT groups postintervention showed nonsignificant differences (*p* > 0.05) through post hoc analysis as demonstrated in Table 2.

## 4. Discussion

This study aimed to evaluate the effects of HILT versus LLLT on patients with chronic nsLBP hypothesizing that laser therapy whether LLLT or HILT may relieve pain and improve quality of life in chronic nsLBP patients. The findings of this study showed that both LLLT and HILT result in a significant improvement of ODI, VAS, lumbar ROM, and EuroQol scores at the end of the study intervention.

The analgesic and anti-inflammatory effects of LLLT are associated with elevating of pain threshold and inhibiting the transmission of A-δ and C fibers that combined with suppressed peripheral nociceptors, increased hydroxyindoleacetics, decreased inflammatory cytokines such as IL-1*β*, IL-8, and TNF-*α*, and reduced prostaglandin levels [30]. Moreover, LLLT inhibits pain through enhancing the secretions of peripheral endogenous opioids [31]. It was reported that infrared laser GaAlAs with a wavelength of 808 nm is a safe and effective modality for decreasing the disability and reducing the severity of pain in LBP patients [32].

Reducing the level of C-reactive protein with laser irradiation is also associated with antiseditious influences of low-level laser therapy [33]. This study utilizes a high-intensity level therapy instrument, which is a novel manner of laser therapy that recently began. This modality of laser intervention demonstrates similar influences of the common laser while with more focused and powerful photoenergy effects, with a more sufficient concentration of endogenous chromophores throughout the treatment program depending on the wavelength. The proliferation of the laser irradiation through the body realizes easily deep penetration, achieves the spread out in the tissue, enhances the oxidative response of mitochondria, generates ATP, RNA, and DNA, enhances photobiological impacts on the affected tissues, stimulates collagen production of the muscle tendons, and accordingly improves the status of chronic nsLBP patients and their daily life activities [19, 34].

Additionally, the study results showed that patients in laser groups displayed a higher degree of improvement utilizing ODI, VAS, lumbar flexion ROM, and quality of life when compared with the control group. HILT of analgesic and detumescence facilitates inflammation resolution of the local issue, improves circulation, reduces LBP, and improves the recovery of function, coinciding with previous study results. HILT conducts actual-duration, complex, larger scale, deeper penetration, and more dose behavior on the body's affected tissues compared to low-level lasers. The laser therapy also improves disease conditions, which play a key role in relieving pain and function recovery [35].

Although this randomized study provides important findings, it has some limitations. Because the study has included only chronic nsLBP patients with 25–40 years, it cannot be generalized to other ages. Another limitation is the lack of long-term follow-up of the study (6 months to one year postintervention). Future studies may compare laser therapy and other modalities of electrotherapy in the rehabilitation field.

## 5. Conclusions

Based on the study outcomes, there are no different effects of LLLT versus HILT in the treatment of chronic nsLBP. Both LLLT and HILT reduce pain and disability and improve lumbar ROM and quality of life in patients with chronic nsLBP.

## Figures and Tables

**Table 1:** Baseline characteristics of three study groups.

Variables	LLLT (*n* = 20)	HILT (*n* = 20)	Control (*n* = 20)	*p* value
Age, years	32.4 ± 3.7	33.6 ± 4.5	32.8 ± 4.2	0.651
Gender, M/F	14/6	13/7	15/5	0.836
BMI, kg/m^2^	25.4 ± 2.9	25.9 ± 2.5	26.2 ± 2.7	0.642
LBP duration, months	8.12 ± 3.5	7.94 ± 3.2	8.25 ± 3.7	0.961
*Education level, n (%)*				
Academic education	6 (30)	7 (35)	5 (25)	0.913
Middle education	10 (50)	8 (40)	9 (45)	
Primary education or less	4 (20)	5 (25)	6 (30)	
*Occupation, n (%)*				
Employed	10 (50)	9 (45)	11 (55)	0.981
Unemployed	4 (20)	5 (25)	4 (20)	
Workers	6 (30)	6 (30)	5 (25)	
*Marital status, n (%)*				
Married	16 (80)	18 (90)	15 (75)	0.506
Single	4 (20)	2 (10)	5 (25)	

Variables are presented as means ± standard deviations; significant at *p* value <0.05; M: males; F: females; BMI: body mass index; ODI: Oswestry Disability Index; VAS: visual analogue scale; ROM: range of motion; EuroQol: European Quality of Life score.

**Table 2:** The differences in mean values within and between groups pre- and postintervention.

Variables	Preintervention	Postintervention	Mean difference (95% CI)	*p* value
*ODI*				
LLLT	36.5 ± 12.7	17.8 ± 6.4	18.75 (16.8 to 20.6)	<0.001
HILT	37.3 ± 11.3	18.5 ± 7.2	18.8 (16.77 to 20.8)	<0.001
Control	36.2 ± 12.3	34.6 ± 11.8	1.6 (−1.4 to 5.3)	0.395
*p* value	0.726	<0.001	—	—
*VAS*				
LLLT	6.5 ± 1.7	3.4 ± 0.9	3.1 (2.5 to 3.6)	<0.001
HILT	6.3 ± 1.9	3.5 ± 0.8	2.8 (2.4 to 3.2)	<0.001
Control	6.6 ± 1.6	5.9 ± 1.8	0.7 (0.19 to 1.2)	0.293
*p* value	0.534	<0.001	—	—
*Lumbar ROM*				
LLLT	17.3 ± 2.2	20.4 ± 1.7	−3.1 (−2.5 to −3.6)	<0.001
HILT	16.9 ± 2.1	20.6 ± 1.8	−3.7 (−2.8 to −4.1)	<0.001
Control	17.6 ± 2.5	17.9 ± 2.4	−0.3 (−0.39 to −0.21)	0.082
*p* value	0.623	<0.001	—	—
*Total EuroQol*				
LLLT	32.5 ± 8.4	72.8 ± 9.7	−40.3 (−45.6 to −36.7)	<0.001
HILT	35.7 ± 9.6	74.3 ± 10.4	−38.6 (−42.3 to −35.4)	<0.001
Control	35.4 ± 8.8	38.6 ± 9.2	−3.2 (−5.3 to −1.4)	0.079
*p* value	0.463	<0.001	—	—

Variables are presented as means ± standard deviations; significant at *p* value <0.05; ODI: Oswestry Disability Index; VAS: visual analogue scale; ROM: range of motion; EuroQol: European Quality of Life score.

## Data Availability

The data involved in this study are available from the corresponding author upon request, and privacy-related parts of the patient will not be provided.
